# 50048 Closing the cross-institutional referral loop: Assessment of consultation note quality

**DOI:** 10.1017/cts.2021.584

**Published:** 2021-03-30

**Authors:** April Savoy, Amee Sangani, Michael Weiner

**Affiliations:** 1 Purdue School of Engineering and Technology, Center for Health Information and Communication, U.S. Department of Veterans Affairs, Veterans Health Administration, Health Services Research and Development Service CIN 13-416, VA Medical Center, Regenstrief Institute, Inc.; 2 Indiana University School of Medicine; 3 Center for Health Information and Communication, U.S. Department of Veterans Affairs, Veterans Health Administration, Health Services Research and Development Service CIN 13-416, VA Medical Center, Regenstrief Institute, Inc., Indiana University School of Medicine

## Abstract

ABSTRACT IMPACT: Results will inform the design of health information technologies that assess and improve clinicians’ interpersonal communication supporting co-management of care across health institutions. OBJECTIVES/GOALS: Poor communication and co-management of comorbidities during the referral process increase physician workload, patient burden, and safety risks. In this preliminary study, our objective was to understand how consultants’ notes support physician collaboration within and across health care institutions. METHODS/STUDY POPULATION: We reviewed medical records. Accessing the Indiana Network for Patient Care database, consultation notes were randomly selected from four specialties: cardiothoracic surgery, neurology, rheumatology, and oncology. These specialties were identified, in advance, as challenging in interprofessional communication. The notes reviewed were associated with in-person consultations at a medical network in the Midwest from 2016 to 2019, including internal and cross-institutional (i.e., external) referrals. The Quality of Consult Assessment tool was adapted to assess note quality and co-management facilitation. Two researchers reviewed all records independently. A consensus meeting was then held to discuss and resolve discrepancies. RESULTS/ANTICIPATED RESULTS: Medical records of ten patients with comorbidities were reviewed. The mean age was 67 (SD= 12 years); one patient was a child. All consultation notes contained clinical recommendations. Seventy percent of notes referred to explicit consultant responsibilities. Conversely, only one contained explicit responsibilities for referrers. Medical records denoted reliance on support staff to send messages among referrers, consultants, and patients via phone calls and facsimile. The use of fax machines to send medical records to referrers was more prominent after cross-institutional consultations. DISCUSSION/SIGNIFICANCE OF FINDINGS: Clinical documentation supported specialty referrals for transitions of care rather than co-management of care. Accessing medical records across institutions contributed to a lack of clinical context, and workflow inefficiencies, when attempting to co-manage clinical care.

